# Estimating Individualized Absolute Risk for Esophageal Squamous Cell Carcinoma: A Population-Based Study in High-Risk Areas of China

**DOI:** 10.3389/fonc.2020.598603

**Published:** 2021-01-08

**Authors:** Yi Shen, Shuanghua Xie, Lei Zhao, Guohui Song, Yi Shao, Changqing Hao, Chen Niu, Xiaoli Ruan, Zhaoping Zang, Rena Nakyeyune, Fen Liu, Wenqiang Wei

**Affiliations:** ^1^Department of Epidemiology and Health Statistics, School of Public Health, Beijing Municipal Key Laboratory of Clinical Epidemiology, Capital Medical University, Beijing, China; ^2^National Central Cancer Registry, National Cancer Center/National Clinical Research Center for Cancer/Cancer Hospital, Chinese Academy of Medical Sciences and Peking Union Medical College, Beijing, China; ^3^Department of Molecular Physiology and Biophysics, Holden Comprehensive Cancer Center, University of Iowa Carver College of Medicine, Iowa City, IA, United States; ^4^Department of Epidemiology, Cancer Institute/Hospital of Ci County, Handan, China; ^5^Department of Endoscopy, Cancer Institute/Hospital of Linzhou, Anyang, China

**Keywords:** absolute risk, individualized, esophageal squamous cell carcinoma, high-risk area, prediction model

## Abstract

**Background:**

Esophageal squamous cell carcinoma (ESCC) has a high incidence rate and poor prognosis. In this study, we aimed to develop a predictive model to estimate the individualized 5-year absolute risk for ESCC in Chinese populations living in the high-risk areas of China.

**Methods:**

We developed a risk-predicting model based on the epidemiologic data from a population-based case-control study including 244 newly diagnosed ESCC patients and 1,220 healthy controls. Initially, we included easy-to-obtain risk factors to construct the model using the multivariable logistic regression analysis. The area under the ROC curves (AUC) with cross-validation methods was used to evaluate the performance of the model. Combined with local age- and sex-specific ESCC incidence and mortality rates, the model was then used to estimate the absolute risk of developing ESCC within 5 years.

**Results:**

A relative risk model was established that included eight factors: age, sex, tobacco smoking, alcohol drinking, education, and dietary habits (intake of hot food, intake of pickled/salted food, and intake of fresh fruit). The relative risk model had good discrimination [AUC, 0.785; 95% confidence interval (CI), 0.749–0.821]. The estimated 5-year absolute risk of ESCC for individuals varied widely, from 0.0003% to 19.72% in the studied population, depending on the exposure to risk factors.

**Conclusions:**

Our model based on readily identifiable risk factors showed good discriminative accuracy and strong robustness. And it could be applied to identify individuals with a higher risk of developing ESCC in the Chinese population, who might benefit from further targeted screening to prevent esophageal cancer.

## Introduction

Esophageal cancer (EC) is a common malignancy with a very poor prognosis. It ranks the seventh most common malignancy and the sixth leading cause of cancer-related deaths among all malignancies worldwide, with an estimated 572,000 new cases and 509,000 deaths in 2018 (1). Esophageal squamous cell carcinoma (ESCC), the predominant histopathologic type of EC, accounts for nearly 90% of all EC cases in lower-income countries, especially in parts of Asia (2). Although the mortality rate of ESCC has been decreased in the last few decades, China is still responsible for more than half of the global burden of ESCC (3). And in China, the incidence and mortality rates of ESCC vary significantly across the country, with over 10-fold differences among regions. In areas around the Taihang Mountains in North Central China, such as Linzhou County and Cixian County, the incidence rate of EC is nearly 100 per 100,000 population (4).

The data collected in the past few years have shown a poor prognosis and survival of ESCC; the overall 5-year survival rate of ESCC in China was approximately only 30.0% (5). One potential strategy to reduce the mortality of ESCC is to identify people at high risk, either preventing the onset of ESCC or increasing the efficiency of large-scale endoscopic screening (6). Our previous studies have shown that the use of endoscopic screening and treatments led to a reduction in ESCC-associated mortality (7). Over the past decade, a series of epidemiologic studies have been conducted in two high-risk areas in China, Linzhou, and Cixian, to investigate the potential risk factors for ESCC. Cigarette smoking, alcohol consumption (8, 9), socioeconomic status, family history of esophageal cancer and eating habits, including the intake of hot foods (10), fruits and vegetables, have been confirmed to be associated with ESCC (11). Using a risk prediction model based on readily identifiable risk factors to identify individuals at the high absolute risk of developing cancer might be a more practical and efficient strategy for cancer prevention. For example, risk assessment tools for breast and colorectal cancers have been applied to inform preventive strategies for these cancers: persons with high-risk scores are encouraged to undergo screening tests (12, 13). Yet, such predictive models for ESCC have been mainly developed for the western populations (14), and have been rarely studied in Asian countries, including China. Due to the significant differences in ESCC between western countries and China, the current models developed in western countries may not be suitable for people living in high prevalence regions of China. In this study, therefore, we aimed to develop a model to determine the individualized absolute risk of ESCC in the next 5 years in a Chinese population, based on a large population-based endoscopic screening trial in Linzhou County of Henan Province and Cixian County of Hebei Province, which were both high prevalence areas of ESCC in China.

## Materials and Methods

### Study Design, Participants, and Data Collection

In 2015, we launched a new screening study of upper gastrointestinal cancer in Linzhou County of Henan Province and Cixian County of Hebei Province. A total of 20,000 participants were enrolled by a stratified cluster sampling procedure and participated in a baseline study on histopathology of the esophageal mucosa. All participants in the screening group were provided endoscopic examination by local physicians who were trained by and under the supervision of experienced doctors from the National Cancer Center in China. The detailed study protocol was described in a previous study (15).

The current study recruited 1,464 participants. They included 244 newly diagnosed ESCC cases: 42 cases from our early screening population, and 202 cases from the Endoscopy Center of Cancer Hospital, Linzhou County, Henan province, between 2014 and 2016. The participants were given careful endoscopic examinations of the entire esophagus and stomach. Then, suspicious biopsy specimens were obtained and read independently by two well-trained local pathologists. The histological diagnostic criteria were as previously described (16). The most severe diagnosis indicated by any of the biopsies was given as the global diagnosis for a participant. All ESCC cases in this study were diagnosed for the first time and pathologically confirmed. The stage and grade of ESCC were assessed according to the 7th edition of the American Joint Committee on Cancer (AJCC) tumor-node-metastasis (TNM) staging system. A total of 1,220 eligible controls were randomly selected from our early 20,000 screening population. All the eligible controls are residents in the selected villages/communities, with no history of cancer or endoscopic examination in the latest 3 years, and are mentally and physically competent. All controls were frequency-matched by age (± 5 years), sex, and residence area to the ESCC cases.

A computer-aided face-to-face questionnaire was completed by each participant to investigate candidate risk factors of ESCC. Information on demographic factors (age at enrollment, sex, and residence area), socioeconomic status (educational level), lifestyle habits (including tobacco smoking, alcohol drinking, and dietary habits), medical history, family history of cancer were collected by trained interviewers. Detailed information on these variables was shown in **Supplementary Material**. All the information about cases and controls collection procedures was similar in design and comparable. This study protocol was approved by the ethics committee of Cancer Institute and Hospital, Chinese Academy of Medical Sciences (approval no. 2015SQ00223), and institutional review board of Capital Medical University (approval no. Z2019SY005), and the written informed consent was obtained from each participant.

## A relative Risk Model for Esophageal Squamous Cell Carcinoma

Variables included in the relative risk model were based on literature review and previous findings of epidemiological studies. And the candidate variables for the inclusion of the model should be easily obtained through questionnaires. We included 10 variables as candidate risk factors for the model, including age, sex, tobacco smoking, alcohol drinking, educational years, family history of upper gastrointestinal cancer, history of upper gastrointestinal disease, intake of hot food, intake of pickled/salted food, and intake of fresh fruit.

We used a two-phase predictor selection method to identify the final panel of variables to be included in the models. First, four variables, age, sex, tobacco smoking, and alcohol drinking, were directly entered into the model with a specified priori from the literature review and associations found in the same case-control study (11, 14). We performed a random forest analysis to evaluate the importance of candidate variables by mean decrease in accuracy score. The candidate variables were included in a sequence according to their importance, from high to low scores. We selected the variables using unconditional logistic regression with a forward selection method. The cut-off for the entry and departure of the logistic regression model was 0.05 and 0.10, respectively. Then, the variables with no significance in univariable logistic regression were included in the multivariable logistic regression model to identify any variables that significantly improved the goodness of fit of the model. We included the cross-product terms and main effect terms in the model to examine any potential interactions between variables. No interaction terms showed statistical significance and thus were removed from the final model. The area under the receiver operating characteristic curve (AUC) was used to evaluate the performance of the relative risk model. Considering the possible overfitting of the model, we performed 10-fold cross-validation and leave-one-out cross-validation to evaluate the generalization error of the predicted probabilities. In the cross-validation process, all data were randomly divided into training set and validation set. The ESCC prediction model was then developed using the training set based on the final panel of variables, and the performance of the model was assessed by validation data. The AUC values were generated using the probabilities calculated from the model for each selected group or participant. All analyses were conducted using SPSS (version 23.0) and R software (version 3.6.2), and all significance tests were two-sided at an α value of 0.05.

### Estimation of Absolute 5-Year Risk of Esophageal Squamous Cell Carcinoma

We estimated the individualized 5-year absolute risk of ESCC from information on the relative risk, the baseline hazard rate and the competing risk (17). Briefly, we calculated the 5-year absolute risk of ESCC for an individual based on the following information: 1) the relative risk of ESCC, which was calculated as the product of the odds ratios (ORs) for the individual with specific risk factors; 2) the estimated population attributable risk (PAR) deriving from the relative risk model (18); 3) the baseline hazard rate that was calculated using local age- and sex-specific ESCC incidence data from the National Central Cancer Registry of China; 4) the local age-specific and sex-specific mortality data excluding ESCC in the population, to correct the risk of death from competing causes. Detailed methods and formulae are provided in the **Supplementary Material**.

## Results

A total of 1464 participants were included in this study: 244 ESCC cases and 1,220 healthy controls recruited in Linzhou County and Cixian County, two high-risk areas of ESCC in China, from 2014 to 2016. The participants were aged between 40 and 74 years, with an average age of 59.5 years, ESCC patients had more individuals with age over 60 compared with the control participants. The male-female ratio is 1.90. Detailed demographic characteristics of all participants and TNM stage of ESCC patients were shown in **Table 1**.

**Table 1:** Characteristics of participants and estimated ORs from the relative risk model.

Variables	ESCC cases n (%)	Controls n (%)	Crude OR (95% CI)	Adjusted OR^*^ (95% CI)
Age, years				
40–49	20 (8.20)	175 (14.34)	1.00	1.00
50–59	66 (27.05)	367 (30.08)	1.57 (0.93–2.68)	1.53 (0.85–2.74)
60–69	124 (50.82)	613 (50.25)	1.77 (1.07–2.92)	1.38 (0.79–2.43)
≥70	34 (13.93)	65 (5.33)	4.58 (2.46–8.52)	2.73 (1.35–5.52)
Sex				
Female	84 (34.43)	420 (34.43)	1.00	1.00
Male	160 (65.57)	800 (65.57)	1.00 (0.75–1.34)	0.74 (0.50–1.09)
Tobacco smoking				
Non-smokers	149 (61.07)	869 (71.23)	1.00	1.00
Smokers	95 (38.93)	351 (28.77)	1.58 (1.19–2.10)	1.27 (0.87–1.87)
Alcohol drinking				
Non-drinkers	188 (77.05)	1125 (92.21)	1.00	1.00
Drinkers	56 (22.95)	95 (7.79)	3.53 (2.45–5.08)	3.75 (2.39–5.88)
Education, years				
≤6	75 (30.74)	675 (55.33)	1.00	1.00
>6	169 (69.26)	545 (44.67)	0.36 (0.27–0.48)	0.38 (0.27–0.54)
Hot food, times/week				
<2	170 (69.67)	1074 (88.03)	1.00	1.00
≥2	74 (30.33)	146 (11.97)	3.20 (2.32–4.42)	2.72 (1.90–3.90)
Pickled/salted food, times/week				
<2	207 (84.84)	1144 (93.77)	1.00	1.00
≥2	37 (15.16)	76 (6.23)	2.69 (1.77–4.09)	2.45 (1.50–3.99)
Fresh fruit, times/week				
<2	184 (75.41)	505 (41.39)	1.00	1.00
≥2	60 (24.59)	715 (58.61)	0.23 (0.17–0.32)	0.25 (0.18–0.34)
Family history of upper gastrointestinal cancer				
No	171 (70.08)	960 (78.69)	1.00	–
Yes	73 (29.92)	260 (21.31)	1.58 (1.16–2.14)	–
History of upper gastrointestinal disease				
No	207 (84.84)	1099 (90.08)	1.00	–
Yes	37 (15.16)	121 (9.92)	1.62 (1.09–2.42)	–
Stage, n (%)				
I	50 (20.49)	–	–	–
II	118(48.36)	–	–	–
III	68(27.87)	–	–	–
IV	8(3.28)	–	–	–

ESCC, esophageal squamous cell carcinoma; CI, confidence interval; OR, odds ratio.

^*^Adjusted for age, sex and other variables in the model.

Detailed information on variables was shown in **Supplementary data**.

### A Relative Risk Model for Esophageal Squamous Cell Carcinoma

Considering the accessibility and accuracy of the variables, we conducted multivariable logistic regression analyses based on the 10 candidate variables in **Table 1**. These candidate variables were included in a sequence according to their mean decrease in accuracy scores (**Figure S1**). After adjusting for other variables, alcohol drinking, frequent intake of hot food and pickled/salted food (≥ 2 times per week) were associated with increased risk of ESCC. Whereas high educational level (> 6 years) and eating fresh fruit frequently (≥ 2 times per week) showed a significant association with decreased risk of ESCC (**Table 1**). The final relative risk model was constructed based on the eight factors including age, sex, smoking, drinking, educational years, intake of hot food, intake of pickled/salted food, and intake of fresh fruit. The distribution of these factors among controls and ESCC cases and estimated ORs with confidence intervals (CIs) were summarized in **Table 1**. More results including model coefficients for the risk prediction model were presented in **Table S1**. The risk factors in the model totally accounted for a PAR of 89.0%.

### Diagnostic Performance of the Relative Risk Model

**Figure 1:**
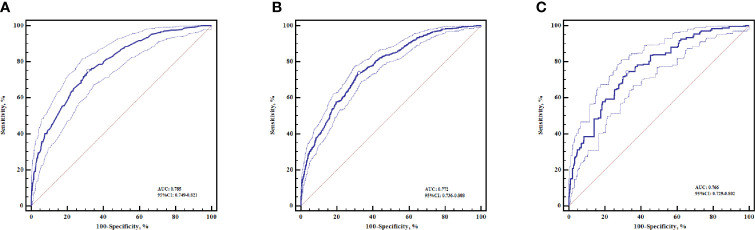
The ROC curves for the relative risk model in discriminating ESCC patients from healthy controls. **(A)** ROC curve without cross-validation **(B)** ROC curve with 10-fold cross-validation **(C)** ROC curve with leave-one-out cross-validation, The optimal cut-off points (dots) were marked on the ROC curves. ESCC, esophageal squamous cell carcinoma. ROC, receiver-operating characteristic curve.

The diagnostic accuracy of the relative risk model in discriminating against the ESCC patients and healthy controls was evaluated by AUC values. When this model was applied to the case-control population, the AUC of the model was 0.785 (95% CI: 0.749–0.821) (**Figure 1A**). In order to obtain more reliable results, we also used 10-fold cross-validation and leave-one-out cross-validation to assess the performance of the model. The AUC obtained from 10-fold cross-validation was 0.772 (95% CI: 0.736–0.808) (**Figure 1B**). A more robust evaluation of AUC from leave-one-out cross-validation was slightly decreased to 0.766 (95% CI: 0.729–0.802) (**Figure 1C**). Cross-validation results indicated that the relative risk model had good diagnostic accuracy and low over-fitting.

### The Absolute 5-Year Risk of Esophageal Squamous Cell Carcinoma

**Figure 2:**
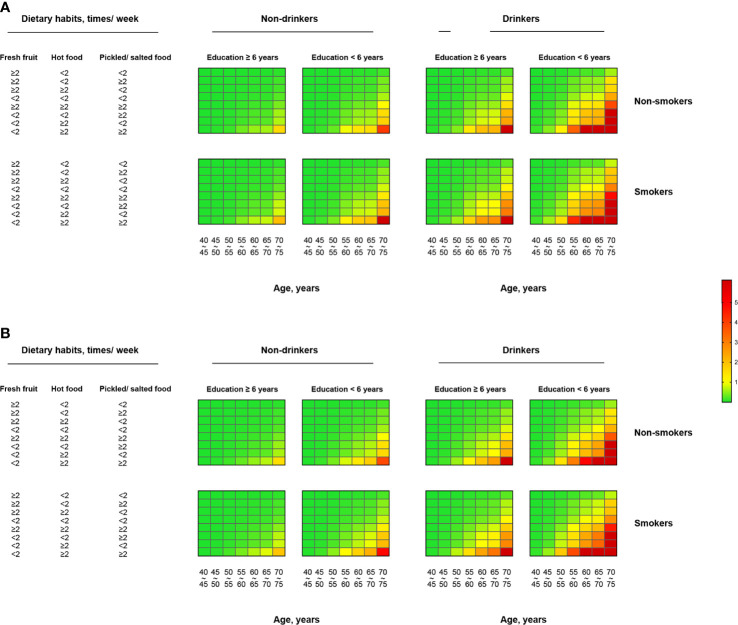
Individualized absolute 5-year risk (%) of ESCC for **(A)** man and **(B)** woman, estimated from the relative risk model and local incidence and mortality rates. ESCC, esophageal squamous cell carcinoma.

**Figure 3:**
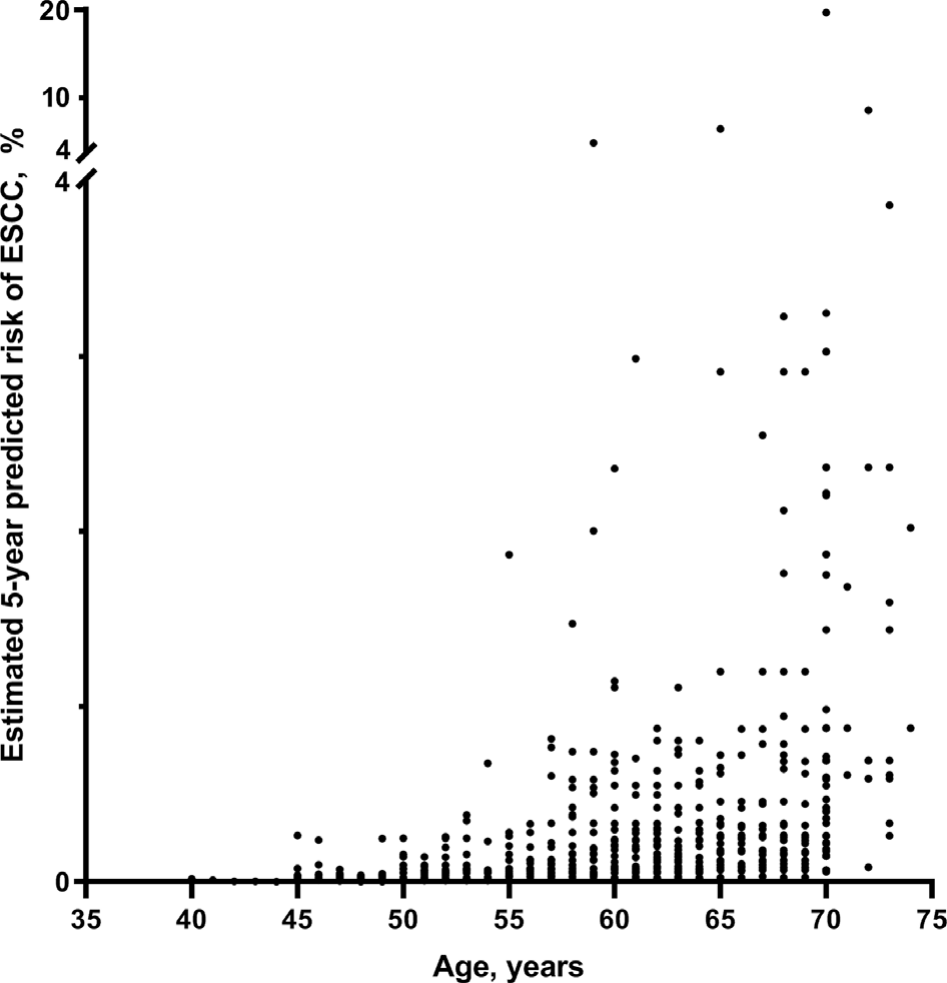
The scatter plot of absolute 5-year risk (%) of ESCC in the studied Chinese population. ESCC, esophageal squamous cell carcinoma.

Based on the relative risk model from the case-control study and ESCC incidence and mortality data (**Table S2**), we estimated the individualized 5-year absolute risk for ESCC. The estimated 5-year risks of ESCC for individuals with different profiles of the risk factors are presented in **Figure 2**. Absolute risks of ESCC differed widely among populations, varying from 0.0005% to 19.72% in men and from 0.0003% to 17.10% in women, depending on age, sex and other exposed risk factors (**Table 2**). For individuals who neither smoke nor drink, risks for ESCC were increased from 0.0005% to 4.516% in men and from 0.0003% to 3.864% in women. ESCC risks for individuals who both smokes and drinks were increased from 0.0024% to 19.72% in men and from 0.0015% to 17.10% in women. The highest estimated absolute risk (19.72%) was found in men aged 70–75 years who smoke and drink, having less education, and poor eating habits (high intake of hot food and pickled/salted food, and low intake of fresh fruit) (**Figure 2A**). It means that in this group, six individuals are needed to be further investigated, for example, by endoscopy or other screening tools, to detect one case of ESCC within 5 years (**Table 2**). Notably, compared with women, absolute risks were slightly higher in men, and the estimated 5-year absolute risks of ESCC were increased with age in our studied population (**Figure 3**).

**Table 2:** Estimated 5-year absolute risk of ESCC in the Chinese population from high prevalence regions with different risk factors.

Profile of risk factors	Age (years)		Sex	Tobacco smoking	Alcohol drinking	Education, years	Hot food,times/week	Pickled and salted food, times/week	Fresh fruit,times/week	5-year absolute risk, %	Individuals needed to investigate to identify 1 case
1	40–45		Male	Non-smokers	Non-drinkers	>6	<2	<2	≥2	0.0005	198,698
2	40–45		Male	Smokers	Drinkers	>6	<2	<2	≥2	0.0024	41,722
3	50–55		Male	Non-smokers	Non-drinkers	≤6	<2	<2	≥2	0.0131	7,647
4	60–65		Male	Smokers	Drinkers	>6	<2	<2	≥2	0.1059	945
5	60–65		Male	Smokers	Drinkers	≤6	<2	<2	≥2	0.2785	360
6	60–65		Male	Smokers	Drinkers	≤6	≥2	≥2	<2	7.1621	14
7	70–75		Male	Non-smokers	Non-drinkers	≤6	≥2	≥2	<2	4.5162	23
8	70–75		Male	Smokers	Drinkers	≤6	≥2	≥2	<2	19.7225	6
9	40–45		Female	Non-smokers	Non-drinkers	>6	<2	<2	≥2	0.0003	319,537
10	40–45		Female	Smokers	Drinkers	>6	<2	<2	≥2	0.0015	67,095
11	50–55		Female	Non-smokers	Non-drinkers	>6	<2	<2	≥2	0.0054	18,353
12	60–65		Female	Non-smokers	Non-drinkers	>6	<2	<2	≥2	0.0193	5,186
13	60–65		Female	Non-smokers	Non-drinkers	>6	≥2	≥2	<2	0.5128	196
14	60–65		Female	Smokers	Drinkers	≤6	≥2	≥2	<2	6.2386	17
15	70–75		Female	Non-smokers	Non-drinkers	≤6	≥2	≥2	<2	3.8644	26
16	70–75		Female	Smokers	Drinkers	≤6	≥2	≥2	<2	17.1013	6

ESCC, esophageal squamous cell carcinoma.

## Discussion

The present study developed a prediction model based on information about easily obtainable risk factors, to estimate an individual’s absolute 5-year risk of ESCC. Our model presented good accuracy and robust performance in discriminating ESCC patients from healthy controls (**Figure 1**). To the best of our knowledge, we first developed an individualized absolute risk prediction model of ESCC for the Chinese population. The prediction model included key demographic factors (age and sex), socioeconomic status (educational years), and lifestyle habits (tobacco smoking, alcohol drinking, and dietary intakes) (**Table 1**). The estimated absolute 5-year risks of ESCC among the population varied greatly across the exposure to different risk factors and depended heavily on age and sex (**Figure 2**).

In our model, we found that alcohol drinking, educational years and eating habits were closely related to the incidence of ESCC, even after adjusted for other factors. There are some researches revealing that educational level, often used as an indicator of socioeconomic status, might have an inverse association with the risk of ESCC (9, 19). The intake of hot food and beverages has been shown to be related to increased risk of ESCC in former epidemiological studies (10). Pickled foods were an indispensable part of the diet in many families of high risk areas of China. N-nitroso compounds and mycotoxins could be generated during the pickling process, which contribute to the development of ESCC (20). According to our results, frequent consumption of fresh fruit seems to be a protective factor of ESCC, which was in accordance with those of previous studies (21, 22). Consumption of tobacco is a major risk factor of ESCC in western developed countries (23–25). But, in our population, the effect of smoking on ESCC was relatively weak after adjusted for other factors. The similar mild association between smoking and ESCC was also found in previous prospective studies carried out in China and other economically underdeveloped countries (8, 9). Alcohol consumption was showed to be a risk factor of ESCC in our study. Although alcohol drinking is widely accepted as a major risk factor of ESCC, evidence showed that its effect varied greatly among regions with different incidence rates and largely depended on the doses of alcohol intake (11). The relationship between alcohol drinking and risk of ESCC was still unclear, and further investigation is needed in high-risk areas of rural China.

Several studies have focused on predicting the risk of ESCC. A risk prediction model combining known lifestyle factors with genetic variants has been developed for identifying ESCC patients in the Chinese population (26). Age-stratified risk prediction models were also established based on a large-scale population to distinguish ESCC and its malignant precancerous lesions (severe dysplasia) from healthy people (27). However, those models did not estimate individuals’ absolute risks and therefore differed in form and function from our model. Moreover, compared to the model developed on a hospital-based case-control study (28), our model from the large population-based study was established on different variables including dietary habits, and it presented good discrimination accuracy. A recent model for the Sweden population contains six variables: age, sex, tobacco smoking, alcohol overconsumption, education and duration of living with a partner, and place of residence during childhood (14). Several factors included in our model were same as the factors in the Sweden model (including age, sex, smoking, and drinking status), indicating these factors might play essential roles in the development of ESCC. Further, due to the differences in race, environment and socioeconomic factors, the prevalence and risk factor profiles for ESCC are quite different between western countries and Asian developing countries. It is necessary to develop specific prediction models based on risk factors of different populations to improve the accuracy and performance of the predictive models for ESCC.

Moreover, the risk model in this study could be applied to quantify the individual risk for ESCC and have public health implications for ESCC prevention in high-risk areas of China. On the one hand, the decision to receive endoscopy examination or other invasive investigations could be made based on the absolute risk assessment of developing ESCC within 5 years, which can be achieved by questionnaires. For example, in this high risk area, for a 40-year-old woman with no exposure to any risk factors, her absolute 5-year risk for ESCC is 0.0003%. This low absolute risk would suggest sparing her from unnecessary, costly, and invasive investigations. In contrast, a 70-year-old man exposed to all risk factors has an estimated 5-year risk for ESCC up to 19.27%. Such a high absolute risk would strongly recommend him to adopt endoscopy detection. On the other hand, it is extremely costly and difficult to carry out a universal endoscopic screening even in areas with a high incidence of ESCC. Identifying individuals at high risks could greatly increase the efficiency and rationalize resource distribution for ESCC screening. According to our study, in the low-risk group, 319,537 individuals are needed to investigate by endoscopy examination to detect one case of ESCC within 5 years. While in the high-risk group, diagnosing one ESCC patient required endoscopy in only six individuals (**Table 2**). Therefore, the model developed in this study might be helpful in planning professional screening programs and making public health decisions, as well as in targeting future interventions for the prevention of ESCC.

Like other risk predictive analyses (29, 30), a limitation of our study was that the absolute risk model is developed based on a case-control study, though it is a population-based study with a relatively large sample size. Furthermore, any generalization of our model to other populations in high incidence areas should be concerned. We performed internal validation using the leave-one-out and 10-fold cross-validation methods to assess the diagnostic accuracy of the model, but external validation in independent populations is necessary. And the ongoing prospective, randomized controlled screening trial in China might provide an opportunity to further validate and refine the model in an independent cohort.

In conclusion, our study developed a model using readily identifiable risk factors, to estimate the individualized 5-year absolute risk of ESCC in the population from high prevalence areas of ESCC in China. Our model has the potential to identify individuals with high risk of ESCC and might be useful for ESCC prevention in high-risk areas of China. However, to confirm the universality of the model, prospective studies with large-scale validation populations are necessary.

## Data Availability Statement

Detailed information support of the study is available in the **Supplementary Material**.

The data that support the findings of this study are available on request from the corresponding authors. Requests to access the datasets should be directed to WW, weiwq@cicams.ac.cn; FL, liufen05@ccmu.edu.cn.

## Ethics Statement

The studies involving human participants were reviewed and approved by the ethics committee of Cancer Institute and Hospital, Chinese Academy of Medical Sciences (approval no. 2015SQ00223), and institutional review board of Capital Medical University (approval no. Z2019SY005). The patients/participants provided their written informed consent to participate in this study.

## Author Contributions

FL and WW conceptualized and designed the study. YShe and FL developed the methodology. SX, GS, and CH acquired the data (provided animals, acquired and managed patients, provided facilities, etc.). YShe, YSha, CN, and XR analyzed and interpreted the data (e.g., statistical analysis, biostatistics, computational analysis). YShe, FL, LZ, and WW wrote, reviewed, and/or revised the manuscript. ZZ and RN gave administrative, technical, or material support (i.e., reporting or organizing data, constructing databases). FL and WW supervised the study. All authors contributed to the article and approved the submitted version.

## Funding

This work was supported by grants from the National Natural Science Foundation of China (81874277 and 81473056), the National Key R&D Program of China (2016YFC0901400 and 2016YFC0901404), Ministry of Science and Technology of China (201502001).

## Conflict of Interest

The authors declare that the research was conducted in the absence of any commercial or financial relationships that could be construed as a potential conflict of interest.
