# Supplementary material for: Estimating Individualized Absolute Risk for Esophageal Squamous Cell Carcinoma: A Population-Based Study in High-Risk Areas of China
Source: Front Oncol. 2021 Jan 8;10:598603. doi: 10.3389/fonc.2020.598603 (PMC7821851; doi:10.3389/fonc.2020.598603)
Supplement: Supplementary file 1 [file DataSheet_1.doc]

***Supplementary Material***

# 1 Supplementary Data

**1.1 Detailed information of variables**

(1) Age

Age at enrollment was calculated as the following: Age=date (interview) - date (birthday).

The age of each individual was treated as a categorical variable: 40~49 years old (coded as 0), 50~59 years old (coded as 1), 60~69 years old (coded as 2) and age ≥ 70 years old (coded as 3).

(2) Sex

Females are coded as 0 and males are coded as 1.

(3) Tobacco Smoking status

Subjects were asked about their lifetime history of tobacco smoking. It includes two statuses: non-smokers (coded as 0) and smokers (coded as 1). Smokers refer to continuous smoking for more than six months with a self-estimated daily consumption of at least one cigarette. The non-smokers have no current or previous history of tobacco use.

(4) Alcohol drinking status

Subjects were asked about their lifetime history of alcohol drinking. Alcohol drinking includes two statuses: non-drinkers (coded as 0) and drinkers (coded as 1). Drinkers were defined as drinking at least one drink per day for more than 1 year.

(5) Educational years

Subjects were asked about the years of their education and were categorized into 2 groups as education years ≤ 6 (coded as 0) and education years > 6 (coded as 1).

(6) Dietary Habits

1. Intake of hot food. This variable was categorized into 2 groups: (a) eating sometimes (< 2 times/ week) was coded as 0, and (b) eating frequently (≥ 2 times/ week) was coded as 1.
2. Intake of pickled/salted food. This variable was categorized into 2 groups: (a) eating sometimes (< 2 times/ week) was coded as 0, and (b) eating frequently (≥ 2 times/ week) was coded as 1.
3. Intake of fresh fruit. This variable was categorized into 2 groups: (a) eating sometimes (< 2 times/ week) was coded as 0, and (b) eating frequently (≥ 2 times/ week) was coded as 1.

(7) Family history of upper gastrointestinal cancers

Subjects were asked about the history of cancers in the immediate family members and relatives within 3 generations. Individuals with at least one relative diagnosed with upper gastrointestinal cancers (including esophageal cancer and gastric cancer) were defined as having a family history of upper gastrointestinal cancer. The participants were categorized into two groups: no (coded as 0) and yes (coded as 1).

(8) History of upper gastrointestinal disease

Subjects were asked about the history of upper gastrointestinal disease, included a history of reflux, esophagitis, gastritis, and gastric or duodenal ulcer. The participants were categorized into two groups: no (coded as 0) and yes (coded as 1).

**1.2 Estimation of absolute 5-year risk of ESCC**

We calculated the absolute 5-year risk for all possible profiles of risk factors, based on the following information:

(1) The relative risk for the individual

The relative risk associated with a specific profile of risk factors was calculated as the product of the odds ratios for individual risk factors.

(2) Baseline age- and sex-specific incidence rates

We obtained the age- and sex-specific incidence rates of ESCC from the National Central Cancer Registry of China (NCCR), which are presented in the supplementary table.

(3) Population attributable risk of the model

The population attributable risk (PAR) of the model was calculated by the following formula:

$$PAR=1-\frac{1}{n}\sum_{i=1}^{n} \left(\frac{1}{r_{i}}\right)$$

where *n* was the number of ESCC cases, *r_i_* was the relative risk for the *i*th case estimated from the logistic regression model (*Bruzzi et al. Am J Epidemiol 1985;122:904-913*).

(4) The age- and sex-specific mortality rates excluding ESCC

We calculated the age- and sex-specific mortality rates excluding ESCC using the population mortality data from NCCR.

For an individual with the age of *t* (in five-year groups), sex of *s* (1=male, 2=female), and relative risk of *r*, we first calculated the baseline hazard as:

$$h_{1(t,s)}=IR_{(t,s)}\times\left(1-PAR\right)$$

where *IR(t,s)* was the age- and sex-specific incidence rate of ESCC in the population.

We estimated the absolute risk of ESCC over 5 years as:

$$P\left(t,s,r\right)=\frac{h_{1(t,s)}r}{h_{1(t,s)}r+h_{2(t,s)}}\times\left(1-e^{-5(h_{1(t,s)}r+h_{2(t,s)})}\right)$$

where *h_2_(t,s)* was the age- and sex-specific mortality rate from competing causes (excluding ESCC).

**2 Supplementary Figures and Tables**

**2.1 Supplementary Figures**

**
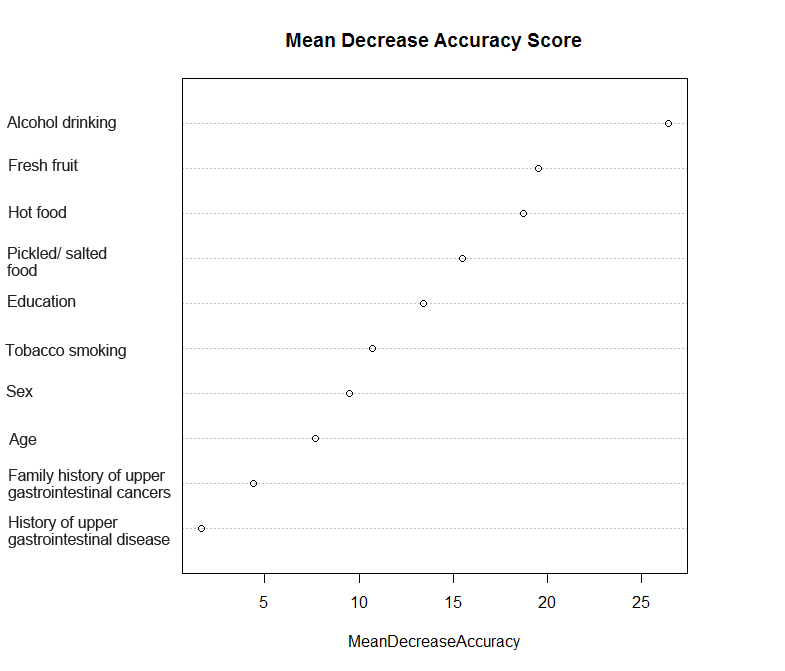
**

**Figure S1.** Mean decrease in accuracy scores of the candidate variables.

**2.2 Supplementary Tables**

| **Table S1.** Parameters of the multivariable logistic regression model | | | | |
| --- | --- | --- | --- | --- |
| **Variables** | **ESCC cases**  **n (%)** | **Controls**  **n (%)** | **Regression coefficient*** | ***P* value** |
| Age, years |  |  |  | 0.023 |
| 40-49 | 20 (8.20) | 175 (13.34) | Reference |  |
| 50-59 | 66 (27.05) | 367 (30.08) | 0.42 |  |
| 60-69 | 124 (50.82) | 613 (50.25) | 0.32 |  |
| ≥70 | 34 (13.93) | 65 (5.33) | 1.01 |  |
| Sex |  |  |  | 0.13 |
| Female | 84 (34.43) | 420 (34.43) | Reference |  |
| Male | 160 (65.57) | 800 (65.57) | -0.30 |  |
| Tobacco smoking |  |  |  | 0.22 |
| Non-smokers | 149 (53.69) | 869 (68.94) | Reference |  |
| Smokers | 95 (23.36) | 351 (23.28) | 0.24 |  |
| Alcohol drinking |  |  |  | <0.001 |
| Non-drinkers | 188 (7.38) | 1125 (2.29) | Reference |  |
| Drinkers | 56 (15.57) | 95 (5.49) | 1.32 |  |
| Education, years |  |  |  | <0.001 |
| ≤ 6 | 75 (30.74) | 675 (55.33) | Reference |  |
| > 6 | 169 (69.26) | 545 (44.67) | -0.97 |  |
| Hot food, times/ week |  |  |  | <0.001 |
| < 2 | 170 (69.67) | 1074 (88.03) | Reference |  |
| ≥ 2 | 74 (30.33) | 146 (11.97) | 1.00 |  |
| Pickled/ salted food, times/ week |  |  |  | <0.001 |
| < 2 | 207 (84.84) | 1144 (93.77) | Reference |  |
| ≥ 2 | 37 (15.16) | 76 (6.23) | .89 |  |
| Fresh fruit, times/ week |  |  |  | <0.001 |
| < 2 | 184 (75.41) | 505 (41.39) | Reference |  |
| ≥ 2 | 60 (24.59) | 715 (58.61) | -1.40 |  |

ESCC, esophageal squamous cell carcinoma

*Regression coefficients were calculated to denote the contribution of each variable in the risk model.

| **Table S2.** The age-specific and sex-specific incidence rates of ESCC and mortality rates (excluding ESCC) per 100,000 in Linzhou County and Cixian County in China, 2014. | | | | | |
| --- | --- | --- | --- | --- | --- |
| **Age, years** | **Man** | |  | **Woman** | |
|  | **Incidence** | **Mortality** |  | **Incidence** | **Mortality** |
| 40~45 | 13.03 | 41.99 |  | 6.00 | 56.98 |
| 45~50 | 50.39 | 96.21 |  | 30.73 | 86.04 |
| 50~55 | 84.43 | 195.71 |  | 68.31 | 86.77 |
| 55~60 | 253.69 | 419.98 |  | 147.02 | 212.59 |
| 60~65 | 424.17 | 675.79 |  | 269.78 | 344.48 |
| 65~70 | 461.79 | 930.33 |  | 354.98 | 395.72 |
| 70~75 | 643.52 | 1210.58 |  | 399.68 | 579.82 |

ESCC, esophageal squamous cell carcinoma.

Note: data were obtained from the National Central Cancer Registry of China (NCCR).
